# What can we learn from inactivation studies? Lessons from auditory cortex

**DOI:** 10.1016/j.tins.2021.10.005

**Published:** 2022-01

**Authors:** Zuzanna A. Slonina, Katarina C. Poole, Jennifer K. Bizley

**Affiliations:** 1 Ear Institute, University College London, London WC1X 8EE, UK

**Keywords:** silencing, optogenetics, lesions, behaviour, hearing, causal manipulation

## Abstract

Inactivation experiments in auditory cortex (AC) produce widely varying results that complicate interpretations regarding the precise role of AC in auditory perception and ensuing behaviour. The advent of optogenetic methods in neuroscience offers previously unachievable insight into the mechanisms transforming brain activity into behaviour. With a view to aiding the design and interpretation of future studies in and outside AC, here we discuss the methodological challenges faced in manipulating neural activity. While considering AC’s role in auditory behaviour through the prism of inactivation experiments, we consider the factors that confound the interpretation of the effects of inactivation on behaviour, including the species, the type of inactivation, the behavioural task employed, and the exact location of the inactivation.

## Highlights

Wide variation in the outcome of auditory cortex inactivation has been an impediment to clear conclusions regarding the roles of the auditory cortex in behaviour.
Inactivation methods differ in their efficacy and specificity. The likelihood of observing a behavioural deficit is additionally influenced by factors such as the species being used, task design and reward.
A synthesis of previous results suggests that auditory cortex involvement is critical for tasks that require integrating across multiple stimulus features, and less likely to be critical for simple feature discriminations.
New methods of neural silencing provide opportunities for spatially and temporally precise manipulation of activity, allowing perturbation of individual subfields and specific circuits.

## Challenges in defining auditory cortex function

Since the location of auditory cortex (AC) in the primate brain was first characterised in 1875 [1], researchers have been attempting to understand its role in auditory behaviours. Initially, the leading method of studying its function was through lesioning different areas of AC in animal models and observing the resulting impairments, with the aim of identifying behaviours for which AC is necessary. Later on, most investigations of AC function became centred on recording cortical activity associated with specific stimuli and behaviours, shifting the focus to correlational studies of neuronal activity and sensory stimuli. While electrophysiological recordings indicate the involvement of AC in nearly all auditory behaviours, from representing basic acoustic properties to associative learning [2], the results of silencing studies vary greatly. However, recent advances in available inactivation methods offer increasingly more spatially and temporally precise manipulations (Table 1).

**Table 1:** Methods of inactivation

Method	Mechanism	Penetrance	Characteristics
Lesions	Irreversible removal of neural tissue [87]	High Dependent on how completely the target brain area is removed [88]	Compensatory plasticity [88,89] Damage to fibres of passage^a^ [90] Degeneration of upstream areas (e.g., thalamus) [88]
Pharmacological	Activation of inhibitory neurons via reagents [82]	Moderate Dependent on ligand diffusion [82]	Area of effect relies on diffusion of reagent which may vary between reagents (e.g., muscimol spreads maximally and γ-aminobutyric acid [GABA] minimally) [82] Difficult to apply to certain brain areas
Cooling	Reduction of cortical temperature to reduce spiking [21]	Moderate Dependent on temperature conduction through tissue [7,8]	Slow but sustained control of inactivation [88] Area of effect is dependent on the size of the cooling loop/probe [21] Can cool nontarget areas via cooled blood vessels [21]
Chemogenetics	Activation of receptors, genetically expressed in target neurons, via ligands [9]	Low to moderate Dependent on delivery, either viral vector or transgenic animals [23,81]	Area of effect is dependent on both diffusion of ligand and expression of receptor [79]
Optogenetics	Activation of photoreceptors, genetically expressed in target neurons, via light [79]	Low to moderate Dependent on delivery, either viral vector [91] or transgenic animals [79] Differential expression between species [79]	Rapid control of inactivation [92] Area of effect is dependent on both spread of light delivery and expression of receptors [79] Cell specificity mostly restricted to mouse model [93] Controls needed against heat generation from light application [92]

a Excitotoxic lesions which spare fibres of passage.

**Figure 1:**
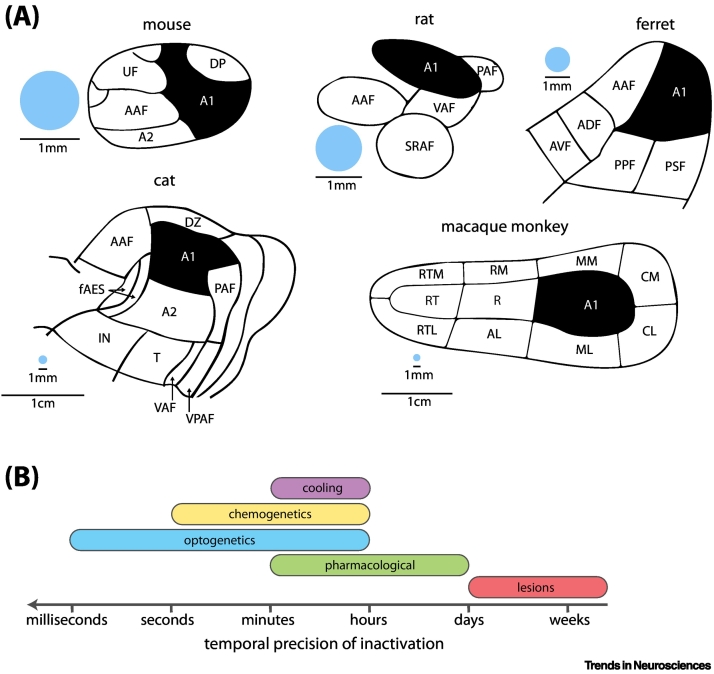
Schematic of auditory cortex in different species and overview of various inactivation methods. (A) Schematics of auditory cortex in the mouse [84], rat, ferret, cat and macaque (modified, with permission, from [85]) with core (primary) areas shaded in black and scale bars indicating 1 mm and in the macaque and cat both 1 mm and 1 cm. The blue circle indicates a diameter of 1 mm relative to each cortex. (B) A schematic displaying the temporal precision of each of the inactivation methods listed: optogenetics (milliseconds to hours if using step-function opsins [79]), chemogenetics (minutes to hours [80]), cooling (onset activation within several minutes, recovery of firing rates up to an hour [81]), pharmacological inactivation (within minutes to several days [82]), and lesions (permanent inactivation postsurgery [83]). Abbreviations: A1, primary auditory field; AAF, anterior auditory field; A2, secondary auditory field; ADF, anterior dorsal field; AL, anterolateral belt; AVF, anterior ventral field; CM, caudomedial belt; CL, caudolateral belt; DP, dorsoposterior field; FAES, auditory field of the anterior ectosylvian sulcus; IN, insular region; ML, mediolateral belt; MM, mediomedial belt; PAF, posterior auditory field; PPF, posterior pseudosylvian field; PSF, posterior suprasylvian field; R, rostral field; RM, rostromedial belt; RT, rostral temporal field; RTL, rostrotemporal lateral; RTM, rostrotemporal medial belt; SRAF, suprarhinal auditory field; T, temporal region; VAF, ventral auditory field.

This, combined with our improved understanding of the functional anatomy of AC and its subregions (Figure 1A), puts us in a better position to revisit inactivation as a method for investigating cortical function in the context of auditory cortex. We will start by considering the factors that influence the likelihood of observing an inactivation-induced deficit whilst bringing together the current knowledge of behavioural effects of AC inactivation, with the goal of guiding future research. Finally, we will attempt to synthesise the existing literature to identify functions in which auditory cortical activity is most strongly implicated.

### Methods for inactivating (auditory) cortex

Approaches to silencing activity in auditory cortex range in timescale from permanent lesions to reversible inactivation via thermal, pharmacological or chemogenetic means (allowing inactivation from minutes to hours) and optogenetics (enabling millisecond precision, Figure 1B). All inactivation methods are subject to the trade-off between certainty that the entire region of interest is inactivated and the risk of inactivating adjacent areas potentially producing off-target effects. Inactivating neural tissue beyond the border of the target area can result in inflating the impairment that would result from a more focal inactivation. Conversely, it has been shown that sparing portions of the target area can be sufficient to subserve some aspects of the studied behaviour [3], creating the risk of underestimating the role of the inactivated structure.

**Figure 2:**
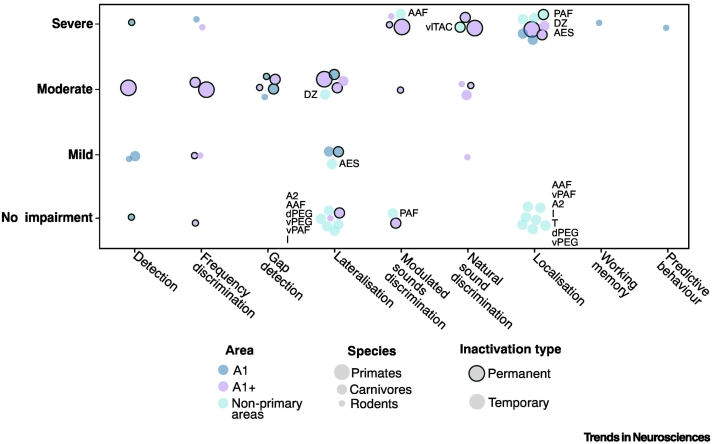
Summary of AC inactivation studies and the observed impairments. Studies in which non‐primary areas were inactivated are labelled with the specific subregion of AC targeted in the study. See also Table 2. Abbreviations: A2, secondary auditory cortex; AAF, anterior auditory field; AES, anterior ectosylvian sulcus; dPEG, dorsal posterior ectosylvian gyrus; DZ, dorsal zone; I, insular region; PAF, posterior auditory field; T, temporal region; vlTAC, ventrolateral temporal region auditory cortex; vPAF, ventral posterior auditory field; vPEG, ventral posterior ectosylvian gyrus.

With such differences in the ability to manipulate inactivation spatially and temporally, each method has its own advantages and limitations (Table 1). Timescale itself is a significant factor since the duration of loss of AC function (Figure 2) shapes the magnitude of the observed deficit: in many cases, performance on a task continues to evolve for a long time after the inactivation, with most severe impairments occurring in the short term [4., 5., 6.]. It has been argued that the ability to recover from a permanent lesion is a hallmark of an area with a **permissive role** (see Glossary) in behaviour, rather than an **instructive** **role** [7]. Thus, contrasting the effects of permanent and temporary inactivation can distinguish between these two possibilities [8]. Nonetheless, given the scope of plasticity within both brain and behaviour, it seems likely that the role of initially instructive areas could be taken on by other circuits. Adaptation itself can result from processes including recovery from surgery [9,10], **adaptive plasticity** [11], relearning the discrimination (i.e., recovering from a memory deficit [2]) or learning to solve the task in an alternative way, either by using different cues [12,13] or by recruiting alternative neural circuits [14,15].

Permanent lesions are likely to have profound effects on areas upstream and downstream of the lesioned site; indeed, retrograde degeneration of the ventral division of the medial geniculate body (MBGv) is a hallmark of a successful A1 lesion [16]. Thus, the behavioural effects of lesioning may lead to overstated conclusions regarding the role of the target region, as in addition to impacts on connected areas, potential damage to blood supply and fibres of passage within the lesioned area may further perturb activity beyond the target [17,18]. By contrast, a reversible inactivation preserves normal activity in between sessions such that connected structures are not severely impacted. During inactivation, it is likely that some residual activity will persist; how that information is interpreted in these structures is unclear. One possibility is that the residual activity is similar enough to physiologically normal activity that it is still treated as viable input, even if it is not informative about the actual sensory stimulus [8,19]. While all reversible inactivation methods are susceptible to being incomplete, improved monitoring of activity in the target area could provide better insight into the extent of inactivation.

Temporary inactivation approaches, especially with a rapid onset, are less susceptible to the confounding effects of plasticity (although some degree of plasticity can occur very rapidly [20]), allowing for clearer insights into functions that the target area is normally involved in, rather than strictly necessary for. Nonetheless, certain aspects of the spatial precision of reversible inactivations are difficult to control. First, both pharmacological agents and cooling may spread to off-target areas [21]. The diffusive properties of pharmacological agents differ (Table 1), but can be partially controlled depending on the method of application: infusions and topical applications offer less control in that regard, while the use of slow-releasing polymers (e.g., Elvax [22]) can help regulate the extent of diffusion. In addition, the efficacy of inactivation through both cooling and use of pharmacological agents may differ across the cortical layers, with more activity remaining in deeper layers [23., 24., 25.].

Compared with lesions and pharmacological methods, optogenetics and chemogenetics potentially offer a much higher degree of spatial precision. Both methods provide the invaluable opportunity to target neuronal activity in a cell-type-specific manner which, in the case of optogenetics, is coupled with millisecond temporal precision [26]. However, there are certain limitations due to the way in which neuronal subtypes are targeted. Notably, there is a risk that rather than fully inhibiting activity in a given area, activity is only reduced and/or perturbed [8]. Furthermore, the method of expression (i.e., viral vectors vs. transgenic expression) will determine the penetrance (lower for viral vectors which is dependent on spread from injection site) and spatial specificity (lower for transgenic animals where the light placement will largely determine specificity) [27]. While histological methods can confirm the placement of virus-mediated expression, the spread of light will also affect the extent of inactivation. Challenges in delivering light – to deeper cortical layers and, in the case of larger species, to a sufficiently large area – mean that the efficacy with which the targeted populations are modulated can be variable [26], likely resulting in incomplete inactivation.

Cell-type-specific manipulations can in certain situations lead to confounding results. In the case of optogenetics, inactivation is usually achieved in one of two ways. One possibility is to activate inhibitory interneurons (most often parvalbumin-positive [PV+] interneurons), which reduce the firing of principal neurons (PN) by providing them with inhibitory input. Alternatively, one can seek to inhibit the firing of excitatory neurons directly. Due to the dense interconnectivity between both PN–PN and PN–PV+ in the cortex, some of the changes resulting from neuron-subtype-specific manipulations might be unexpected. For example, optogenetic suppression of PV+ neurons would be predicted to cause an increase in excitation. However, suppressing PV+ interneurons in layer 4 led to a net increase in PV+ activity in downstream layers [28], indicating that the excitation-inhibition imbalance produced can be corrected within milliseconds from PV+ inactivation. Furthermore, while opsins were expressed in neurons in all cortical layers, the effects of optical illumination suppressed the activity of PV+ neurons only in layer 4. In a similar vein, targeting a specific subtype of neurons defined by its genetic markers can have potential caveats, as different subtypes seem to work as functional units and thus are likely to be tied to specific behaviours [29]. As a result, depending on the subtype of interneurons targeted, the behavioural effects of optogenetic inactivation may differ and may not generalise to the effects of silencing other neuronal subtypes.

### Other factors that influence behavioural deficits

#### Size and location of lesion

The observation that the severity of behavioural impairment scales with the size of the cortical area affected has been made in a number of studies testing different behaviours [22,30,31]. However, electrophysiological and anatomical studies make it clear that AC comprises multiple subregions with different response properties to sounds; therefore, the precise subregion affected should determine the type of deficit that may be observed. Despite this knowledge, the area of inactivation is often ill defined. Most studies to date aimed to inactivate primary AC (A1) as it is most clearly stereotaxically- and cytoarchitectonically-defined and is the region with most well-defined homologues in other species [32]. The hierarchical organisation of auditory cortex means that disrupting primary cortex will additionally perturb processing in non‐primary areas [33,34]. Despite A1 being the most frequently targeted inactivation site, the trade-off between incomplete inactivation within an area and nonspecific effects beyond a target area make it likely that non‐primary auditory fields (Figure 1A) are usually at least partially affected as well, thus obfuscating the exact role of A1. This was particularly well demonstrated in a study in which primary AC and dorsal zone (DZ), a non‐primary area often inactivated in studies targeting primary AC, were inactivated individually, as well as simultaneously [35]. The study showed that the extent of behavioural deficits in sound **localisation** often attributed to loss of function in primary AC is in fact overstated; comparable results can be obtained only when both primary AC and DZ are inactivated simultaneously, but not individually. In larger species silencing distinct regions is more feasible, enabling efforts to identify the particular contributions of specific auditory fields to auditory behaviour [36,37].

#### Species

While formal comparisons of the effects of AC inactivation across species are lacking, there does seem to be systematic variation in the results obtained in studies using primates, carnivores, and rodents (Table 2). It has been speculated that the degree to which auditory perception is dependent on AC is proportional to the acuity of hearing of a given species [24]. For example, sound localisation, which is much less precise in rodents than in carnivores or primates [15], is fully spared in rodents following AC inactivation [38], while in other species it is profoundly impaired [30,37]. Moreover, specificity and precision in localisation of lesions will vary between animals due to differences in size of cortical regions (Figure 1A).

**Table 2:** Degree of impairment on standard auditory tasks following AC inactivation

AC region	Primates	Carnivores		Rodents	
	Sound detection				
	Permanent	Permanent	Temporary	Permanent	Temporary
A1	–	Mild [94]	–	No impairment [89]	Mild (optogenetic upregulation of interneurons, several injections of 20 nl, 1 mm optic fibre [43]) to severe (optogenetic inactivation, volume of injection: 120 nl, size of fibre: 400 μm [58]; topical application of 20 μg of muscimol [24])
A1+	Moderate (partial recovery) [14,95]	Mild [96]	–	–	–
Non-primary areas	–	–	–	–	–
	Frequency discrimination				
	Permanent	Permanent	Temporary	Permanent	Temporary
A1	–	–	–	–	Severe (chemogenetics, 60 nl virus injection)
A1+	Moderate (partial recovery) [40]	Moderate (partial recovery) [96]	–	No impairment [4] to mild impairment [20]	Mild (muscimol: 400 nl at four sites [25]) to severe (topical application of 20 μg of muscimol [24])
Non-primary areas	–	–	–	–	–
	Gap detection				
	Permanent	Permanent	Temporary	Permanent	Temporary
A1	No available studies*	Moderate (partial recovery) [97]	–	Moderate [98]	Moderate (muscimol, 30 μg [99]; optogenetics, 200 μm fibre diameter [100])
A1+		Moderate (partial recovery) [97]	–	Moderate [6,98]	–
Non-primary areas		–	–	–	–
	Lateralisation				
	Permanent	Permanent	Temporary	Permanent	Temporary
A1	–	Mild [41,67] to moderate [3]	Mild (cooling, [35,81])	–	–
A1+	Moderate [12]	Moderate [41]	Moderate [35,81]	No impairment [15,101]	–
Non-primary areas	–	–	No impairment (cooling of AAF, ventral PAF, A2^a^, insular region, temporal region, dorsal posterior ectosylvian gyrus, ventral posterior ectosylvian gyrus [36]) Mild (cooling of anterior ectosylvian sulcus [36]) Moderate (dorsal zone cooling [35])	–	–
	Modulated sound discrimination				
	Permanent	Permanent	Temporary	Permanent	Temporary
A1	–	–	–	–	–
A1+	Severe [40]	No impairment [31]	–	Moderate [4,46] to severe [39]	Severe (optogenetics, 3–5 injections of 200 nl of virus, 400 μm fibre diameter [44])
Non-primary areas	–	–	Severe (cooling of AAF [37]) No impairment (cooling of PAF [37])	–	–
	Natural sound discrimination				
	Permanent	Permanent	Temporary	Permanent	Temporary
A1	–	–	–	–	–
A1+	Severe [14,40]	Severe [102]	Moderate (cooling [47])	Moderate [13,103]	Mild to moderate (optogenetics, upregulation of PV+ interneurons, 200 μm fibre diameter [42])
Non-primary areas	–	Severe (ventral insulo-temporal cortex [102])	–	–	–
	Sound localisation				
	Permanent	Permanent	Temporary	Permanent	Temporary
A1	–	Severe [41,67]	Severe (cooling [35,81])	–	–
A1+	Severe [12,30]	Severe [41,67,94]	Severe (cooling [35,81])	–	–
Non-primary areas	–	–	No impairment (cooling of AAF [37], cooling of AAF, ventral PAF, A2, insular region, temporal region, dorsal posterior ectosylvian gyrus, ventral posterior ectosylvian gyrus [36]) Severe (cooling of PAF or dorsal zone [37]; cooling of anterior ectosylvian sulcus [36])	–	–

^a^ Abbreviation: A2, secondary auditory cortex.

#### Behavioural task design

The design of the behavioural task used in experiments can determine whether or not AC inactivation causes a deficit in performing the tested discrimination. In understanding what sorts of discriminations are affected by inactivation it may be useful to consider simple and **complex discriminations** separately. **Simple discriminations** are those that can be made based only on a single acoustic **feature** and include sound detection, frequency discrimination, and gap detection. Complex discriminations are those that cannot be defined by a single acoustic feature and require integrating across multiple sound frequencies, localisation cues, or over time, and include sound localisation, modulation rate judgements and discrimination of vocalisations. Animal studies typically employ restricted stimulus sets whilst often attempting to tax the discrimination of complex features. Limited or simplified stimulus sets can, without sufficient care, enable seemingly complex tasks to be solvable using low-level, simple features. For example, frequency sweep discrimination (rising vs. falling) is severely impaired when the sweeps cover fully overlapping frequencies, but only mildly affected if the spectra only partially overlapped [39,40]. In the first case, the animal is forced to integrate information across frequencies to establish the direction, whereas in the second case, the first and final frequencies alone can be used to solve the task, without actually having to determine the direction of frequency modulation. When designing paradigms to assess complex discriminations it is often necessary to use a limited stimulus set to successfully train the animal and assess performance. However, it is critical to ensure that the task cannot be solved using a simple feature, such as frequency (for an FM sweep direction task), or the presence of absence of power at a single frequency (for a spectral timbre discrimination task), in place of the complex feature that the task is designed to assess. If the task can be solved using a simple feature, the contribution of auditory cortex may be underestimated by allowing alternative neural circuits to solve the task.

Another factor that can determine the likelihood of eliciting an impairment is the amount of cognitive processing or abstraction required to perform a behaviour. This can be particularly well illustrated with the example of sound localisation tasks which require precise target localisation (approach-to-target) or coarse discrimination between the hemifields from which the sounds originate (left-right **lateralisation**). Approach-to-target tasks require the animal to abstract from a combination of localisation cues and map the sound onto external space, while lateralisation can be solved by knowing only which side of the head the stimulus arose from. In line with this, AC inactivation leads to worse performance on approach-to-target tasks, where these errors still occur within the correct hemifield [37,41].

A final and often overlooked element of task design is the reward contingency associated with inactivated trials or sessions. Inactivated trials can be rewarded with the trained stimulus-response contingency – the advantage of this being that if these trials are identifiable to an animal (which they may be through the sensation of cooling, or the visible laser light) they cannot learn a separate reward contingency. However, providing feedback potentially drives plasticity to maximise the chance that the animal can learn to use alternative strategies or pathways. Alternatively, inactivated trials can be always rewarded, never rewarded, or randomly rewarded with a fixed probability. In these cases, there is less opportunity to train the animal into an alternative strategy. However, if the inactivated trials are discernible to the animal, such an approach runs the risk that animals alter their strategy on such trials, potentially eliciting spurious performance changes [42].

## AC’s role in auditory behaviours

**Figure 3:**
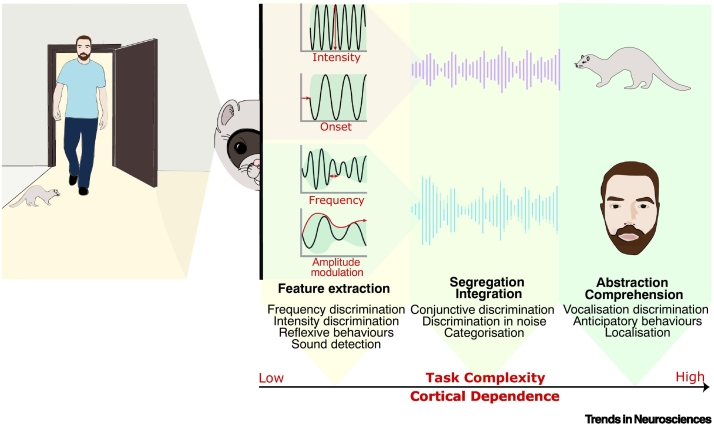
Outline of processing stages involved in auditory scene analysis and associated auditory behaviours. Natural scenes (on the left) consist of a variety of sound sources producing sounds simultaneously. After sounds arrive at the ear, individual acoustic features are extracted, largely at the subcortical level. Accordingly, tasks probing auditory feature extraction are only mildly affected by AC inactivation. The extracted features are then segregated into separate sound sources and integrated into objects defined by conjunctive features. Finally, sounds are interpreted and acted upon, based on their behavioural relevance. The inactivation studies reviewed in the main text support the idea that as task complexity increases through these processing stages, auditory cortex plays an increasingly critical role in successful performance.

Having considered the complexities of designing and interpreting inactivation experiments, we now review AC’s role in auditory perception, where possible considering auditory cortical subfields separately, and when relevant considering differences between rodents, carnivores, and primates. We start by considering simple tasks and move to progressively more complex ones, ultimately arguing that more complex tasks requiring integration over time or frequency, or an additional level of abstraction, are those that are most likely to be consistently impaired by cortical inactivation (Figure 3).

### AC is not necessary for feature-discrimination tasks

In most cases, AC inactivation does not impact the performance of tasks requiring discrimination of simple features. Severe deficits are reported only when reversible inactivation methods are used, that likely target the whole AC [24,43] or for a limited period following a lesion [4., 5., 6.], after which performance gradually recovers. Mild to moderate long-term effects persist for detection/**discrimination thresholds**, and fine discriminations are recovered more slowly than coarse ones after temporary inactivation [24], together suggesting that fine discriminations depend on the AC to a greater extent than coarse discriminations. In summary, AC can provide enhanced acuity for feature discrimination, but its inactivation often leaves such simple discriminations unimpaired [44]. This is consistent with AC acting as a modulator, perhaps shaping the way in which sensory information is processed in the thalamus, which provides sufficient driving input to the structures that guide action (e.g., basal ganglia [104]) to support simple feature discrimination [45].

### AC is required for spectro-temporal integration

The discrimination of complex sounds, such as natural vocalisations, is consistently impaired by AC inactivation. Studies in primates have shown that the ability to discriminate between such sounds is permanently lost in the absence of AC [14,40] (Table 2). Studies testing complex discrimination using artificial sounds such as frequency modulation suggest that this may result from an inability to integrate sound elements over time to group them into a single **auditory object** [46., 47., 48.]. When gerbils were trained to discriminate FM sweeps which differed only in whether the tones were continuously presented or separated by silent gaps, the animals with AC lesions were no longer able to discriminate between these stimuli [46]. Different cortical subfields have been found to be differently involved in temporal integration: in the cat, inactivation of anterior auditory field (AAF) led to severe impairments in discriminating between temporal patterns, while inactivation of posterior auditory field (PAF) led to no impairment [37].

### AC is required for segregation of sound sources

In most listening scenarios, multiple competing sound sources will be present. The brain must process the sound mixture encoded at the ear, appropriately segregating and grouping sound elements in order to reconstruct the underlying sources [49]. Laboratory-based listening tasks rarely employ multiple competing sound sources, preferring simplified stimuli presented in isolation. Nonetheless, impairments consistent with a failure to segregate sounds was demonstrated in a study that tested the ability of ferrets to discriminate vowels either with concurrent noise, continuous noise or in silence. Inactivating AC (at the low-frequency borders of A1 and posterior pseudosylvian field [PPF]/ posterior suprasylvian field [PSF]) led to a substantial performance deficit when the stimuli were presented in noise that shared a temporal onset with the vowel, compared with when vowels were presented in silence, or in continuous background noise, consistent with a difficulty in separating the noise component from the target [47]. Further evidence for the role of AC in source segregation was provided by a study which tested the ability of ferrets to use harmonic cues for sound segregation [48]. Following selective inactivation of auditory corticothalamic fibres, animals were no longer able to detect a mistuned harmonic (which typically ‘pops-out’ of the harmonic complex as a separate sound), highlighting an important role of auditory cortical feedback to the thalamus in segregation [48].

### AC’s role in stimulus abstraction

Auditory cortical neurons can provide invariant representations of perceptual features of sounds [50,51] and can represent category membership [52,53] and behavioural choice [49,54], suggesting that abstraction or categorisation may be a key role of AC. Unfortunately, very few studies have utilised such tasks during AC inactivation. One exception is a flexible categorisation task in which a subject is required to assign pure tones to a ‘high’ or ‘low’ category based on their frequency, where the boundary separating the two categories changes during or across experimental sessions. For sounds near the boundary, the animal must flexibly change the action associated with identical sounds in order to solve the task correctly [55]. Despite this complexity, rodents were found to show only minor impairments in the ability to solve the task following AC inactivation [18,25]. Importantly, in both studies, animals were trained on all possible categorisation contingencies before AC inactivation, leaving the question of whether AC is required for the initial category formation unanswered.

### AC’s role in working memory

Neural correlates of working memory have been observed in auditory cortex [56] and recently optogenetic silencing of auditory cortex in mice revealed a role for AC in an auditory working memory task [57]. In this case, experimenters exploited the temporal resolution of optogenetics and showed that AC activity was shown to be critical for performance in a delayed match to sample task during the presentation of the sound, and also in the early, but not late, delay period [57].

### AC’s role in predicting future acoustic input

Extracting statistical regularities in our environment allows the brain to predict the future behaviour of a given sound source. Echo (or omission) responses are thought to reflect this ability, demonstrated in forming stimulus expectations based on recent sensory experience. While **echo responses** were reliably observed in control mice, no such responses are observed in mice following AC inactivation [58]. When AC was inactivated, the mice were still able to lick in response to a tone, showing they were capable of detecting sound and responding in a stimulus-driven way, but that AC is necessary to form a representation of the rhythmic sound that allows them to form predictions about the upcoming tones. The ability to maintain a representation of a sound source and act accordingly to its expectation is an important aspect of **auditory scene analysis** and object formation, and the AC has been shown to play an important part in this kind of predictive coding [59., 60., 61., 62., 63.].

### AC is required to form an external representation of sound source location

As mentioned earlier, AC inactivation leads to severe impairments in sound localisation in carnivores and primates, but not rodents (Table 2 and Figure 2). Unlike mice and rats, carnivores and primates have access to low-frequency timing cues (interaural time differences), in addition to intensity cues (interaural intensity differences). Precise localisation depends on the integration of binaural cues and monoaural spectral cues in order to determine an unambiguous source location. Lateralisation can be solved based only on a single binaural cue type, by simply determining which ear has a leading interaural time difference or greater **interaural level difference (ILD)**, rather than combining information from both ears to extract the exact difference. Animals such as mice and rats, with access to only one binaural cue type (i.e., ILDs in rats [64] and mice [65,66]), may show less cortical dependency as localisation is arguably a simple feature discrimination in these species.

Studies in carnivores and primates have enabled a more precise investigation into which AC fields underly sound perception. In a foundational series of studies using cortical cooling in cats, a subset of AC fields (A1, DZ, PAF, anterior ectosylvian sulcus [AES]) were shown to be critical for accurate localisation in the cat, whereas cooling others (AAF, A2, ventral PAF) preserved sound localisation behaviour [36,37]. In ferrets, a less clear anterior/posterior distinction is observed: localisation deficits are greater after silencing primary rather than secondary areas, while impairments in adaptation to altered localisation cues are greater when secondary areas are silenced compared with primary ones. In both cases, the magnitude of impairment was equivalent for anterior and posterior fields [41].

Approach-to-target localisation tasks (at least with brief sounds) require an additional level of abstraction: to approach the source requires that the perception of location is externalised (i.e., a source is assigned to some specific position in space). An inability to perceive a locus of sound was proposed in several studies in which AC-lesioned animals were observed to take longer to learn to associate stimuli with responding to the left or the right, as if the association was an arbitrary one (primates [30], ferrets [67]). The need to attribute a sound to an external source requires the ability to switch between an egocentric frame of reference, which is sufficient for lateralisation, and an allocentric frame of reference, which allows for the association between a sound and a location to remain unaltered by the animal’s own movements to solve these approach-to-target localisation tasks. The importance of the ability to switch to an allocentric frame of reference is further highlighted by the fact that animals retain their ability to correctly orient themselves towards the source of sound [68,69] despite their inability to solve approach-to-target tasks. Auditory-evoked motor responses that guide orienting towards the sound are likely initiated in multisensory areas outside of AC, such as the superior colliculus (SC) [70,71] and in primates, frontal eye fields (FEF) [72]. While these mechanisms for localisation seem to operate in parallel to cortical sound localisation and thus remain intact after AC lesions, their output is evidently not sufficient to inform approach-to-target behaviour. This may be due to the reference frame in which location is represented in each of these structures, with, in primates, SC neurons representing an eye-centred perspective [73] and FEF neurons’ activity corresponding to the vector of movement required to face the target, rather than its world-centred location [72]. Neither allow for a stable association between the sound and a locus in space. By contrast, neurons tuned to locations defined in both allocentric and egocentric defined locations have been identified in AC [74], making it a likely candidate to translate between the two frames of reference and attribute sounds to external sources, aiding in auditory object formation.

## Causal manipulations – future perspectives

### Necessity versus sufficiency

Substantiating claims regarding the function of neural structures requires that causal links between neural activity and the behaviour of interest are demonstrated. Inactivation studies, which have been the focus of this review, aim to determine whether the region of interest is indispensable for a particular behaviour. A complementary approach to inactivation studies are so-called “sufficiency” (or “inducing” [75]) experiments. In these experiments, shaping behaviour by stimulating activity in a brain region is taken as evidence that an area plays a causal key role in the behaviour in question. For example, electrical microstimulation has been used to demonstrate functional differences between non‐primary areas in driving a monkey’s decisions in a categorisation task: stimulating cells in AL (but not ML) systematically biased judgements towards the choice associated with the frequency tuning of the stimulation site [53]. Optogenetics offer increased precision in targeting specific neurons in such studies, enabling establishment of direct causal links between specific spatiotemporal patterns of brain activity and behaviour. While most optogenetics studies in auditory cortex to date have focussed on perturbing normal activation patterns to demonstrate a loss of function, an exception is a recent study combining inactivation and stimulation to show that AC is necessary for the discrimination of complex, but not simple sounds and that stimulating AC systematically biased perceptual choices indicating **sufficiency** [44]. Critical to the success of such experiments is the ability to deliver naturalistic patterns of neural activity across populations of neurons [76,77].

### Elucidating circuits

We have already highlighted the paucity of studies targeting individual auditory cortical subfields. A further research direction that offers rich potential is illustrated by studies that selectively inactivated pathways connecting two structures, rather than a whole cortical region or subfield. Such an approach has highlighted the role of corticothalamic feedback in encoding complex features [48], cortico-collicular pathways in driving auditory plasticity [78] and cortico-striatal pathways in guiding auditory decision-making [78]. These examples demonstrate the potential for more nuanced manipulation techniques that allow specific circuits or cell types to be targeted rather than a whole brain region. Such approaches have the potential to reveal the mechanisms involved in driving AC-dependent behaviours, as well as in identifying alternative pathways that could support these tasks in the absence of AC activity.

## Concluding remarks

In this review, we consider the perils and pitfalls of silencing brain activity to assess causal function, with a particular focus on the consequences of auditory cortex inactivation for hearing. Synthesising previous studies, it is clear that AC lesions largely spare the ability to perform tasks that are based on the discrimination of a single sound feature. While some across-species differences are apparent, in general, deficits are particularly evident in tasks that require feature integration or involve discriminating more complex and especially abstract properties of sound (Figure 3). However, when designing and interpreting inactivation studies it is important to remember that the brain is a highly interconnected and plastic system, with each brain region existing within a dynamic network. Thus, the processing required to perform a given auditory behaviour is unlikely to be constricted to a single area, rather, multiple subregions of AC, subcortex, and higher brain regions interact to give rise to a behaviour, with different degrees of involvement. Optogenetic and chemogenetic approaches provide experimenters with opportunities to target pathways or cell types, rapidly and reversibly, in ways that could not be achieved using more traditional techniques. To drive our knowledge of auditory cortex further requires that experimenters exploit these methods while also considering the importance of accurately targeting subregions of auditory cortex (see Outstanding questions). Equally important is the careful design of the stimuli, to ensure they truly tax the behaviour under investigation, and of the behavioural paradigm, to ensure that the experimental subjects cannot solve the task by alternative, simpler, strategies. Putting more emphasis on tasks requiring more advanced cognitive processes, or perception of complex features, could be one of the ways forward in expanding our understanding of the roles of AC.

Outstanding questions What role does AC play in forming auditory scenes and in directing attention to sounds of interest?

What are the consequences of perturbing individual cortical subfields to the tasks that AC has been identified as critical for, and what are the roles of individual cell types within an area identified as playing a role in a given task?

What is the timescale over which behavioural deficits evolve in response to sustained inactivation, and what are the alternative circuits that support performance when AC is silenced?

What is the impact of temporary perturbation of auditory cortical activity on downstream and upstream structures? Can temporary perturbation of auditory cortical activity impair functions that are not supported directly by the AC?

Which impairments following AC inactivation are due to the loss of the processing within AC, and which are due to loss of AC as a relay station?

Alt-text: Outstanding questions

## Glossary

**Adaptive plasticity**: functional reorganisation of brain activity which allows a listener to compensate for functions lost due to damage.
**Auditory object**: a grouping of acoustic features corresponding to a single sound source, or that are perceived as a single event.
**Auditory scene analysis**: the process through which the auditory environment is parsed into separate meaningful units (auditory objects).
**Complex discrimination**: discriminations based on a combination of multiple features.
**Detection threshold**: The minimum magnitude (intensity, duration) that allows for a stimulus to be detected.
**Discrimination threshold**: the minimum difference in a feature that allows two stimuli to be distinguished reliably.
**Echo response**: after a stimulus requiring a certain response is presented rhythmically, eliciting the required response each time, one additional response occurs after the stimulus offset at a time when the next iteration is expected to occur.
**Feature**: in the context of auditory processing, feature refers to any physical property of a sound that is detectable by the auditory system and allows to differentiate between sounds.
**Instructive role**: a brain region has an instructive role in a given process/behaviour if it contributes unique information to that process/behaviour, and therefore its damage causes irreversible loss of function.
**Interaural level difference (ILD)**: the difference in the intensity of the sound perceived in each ear, resulting from the distance between the sound source and each ear.
**Interaural time difference (ITD)**: the difference in time at which a sound arrives at both ears due to the difference in the distance between the sound source and each ear.
**Lateralisation**: discrimination between stimuli presented on the right and on the left of the subject.
**Localisation**: identifying the position of a sound source, including information about both the direction and the distance to the sound.
**Necessity**: property of a brain structure/mechanism whereby its presence is required for a behaviour or cognitive process to occur. Necessity of a brain region can be tested in loss-of-function studies.
**Permissive role**: a brain region has a permissive role in a given process/behaviour if the behaviour/process normally relies on that region, but can be performed by another structure if necessary. Damage to structures with a permissive role will lead to loss-of-function in the short term, which can be recovered over time.
**Simple discrimination**: discriminations based on a simple feature.
**Sufficiency**: property whereby neural activity within a structure induces a behaviour/cognitive process, regardless of other contextual factors.
